# High‐Performance Room Temperature Ammonia Sensors Based on Pure Organic Molecules Featuring B‐N Covalent Bond

**DOI:** 10.1002/advs.202308483

**Published:** 2024-03-14

**Authors:** Qian Wang, Meilong Wang, Kunpeng Zheng, Wanneng Ye, Sheng Zhang, Binbin Wang, Xiaojing Long

**Affiliations:** ^1^ State Key Laboratory of Bio‐fibers and Eco‐textiles Collaborative Innovation Center of Shandong Marine Biobased Fibers and Ecological Textiles Institute of Marine Biobased Materials College of Materials Science and Engineering Qingdao University Qingdao 266071 P. R. China; ^2^ Institute of Nanoscience and Engineering Henan University Kaifeng 475004 P. R. China

**Keywords:** ammonia, B‐N covalent bonds, gas sensor, organic molecules, selectivity

## Abstract

Exploring organic semiconductor gas sensors with high sensitivity and selectivity is crucial for the development of sensor technology. Herein, for the first time, a promising chemiresistive organic polymer **P‐BNT** based on a novel π‐conjugated triarylboron building block is reported, showcasing an excellent responsivity over 30 000 (Ra/Rg) against 40 ppm of NH_3_, which is ≈3300 times higher than that of its B‐N organic small molecule **BN‐H**. More importantly, a molecular induction strategy to weaken the bond dissociation energy between polymer and NH_3_ caused by strong acid‐base interaction is further executed to optimize the response and recovery time. As a result, the **BN‐H**/**P‐BNT** system with rapid response and recovery times can still exhibit a high responsivity of 718, which is among the highest reported NH_3_ chemiresistive sensors. Supported by in situ FTIR spectroscopy and theoretical calculations, it is revealed that the N‐H fractions in **BN‐H** small molecule promoted the charge distribution on phenyl groups, which increases charge delocalization and is more conducive to gas adsorption in such molecular systems. Notably, these distinctive small molecules also promoted charge transfer and enhanced electron concentration of the **P‐BNT** sensing polymer, thus achieving superior B‐N‐containing organic molecules with excellent sensing performance.

## Graphical abstract

A novel strategy to investigate the definite conjugated polymers and sensing activity for chemiresistive‐based organic semiconductor gas sensors by introducing B‐N covalent bond is reported. The as‐fabricated sensing device based on **P‐BNT** exhibited the highest response of 32 000 against 40 ppm of NH_3_ at room temperature. Importantly, the small molecule **BN‐H** with active N‐H can regulate **P‐BNT** to exhibit excellent stability, selectivity, and relative humidity resistance.

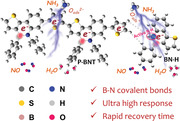

## 1 Introduction

Chemiresistive gas sensors have been widely used to detect hazardous gases and monitor environmental pollution.^[^ ^1^ ^]^ The rapid development of gas sensors benefits from the design of sensitive materials that can respond quickly to specific gases.^[^ ^2^ ^]^ Among various types of gas sensors, ammonia sensors are widely studied because they are easily neglected gas that pollute the environment and pose a great threat to human health.^[^ ^3^ ^]^ Therefore, the design of high‐performance ammonia sensors will facilitate technological advances in environmental analysis,^[^ ^4^ ^]^ indoor air quality control,^[^ ^5^ ^]^ automotive industry,^[^ ^6^ ^]^ and medical applications.^[^ ^7^ ^]^ With the increasing variety of gas‐sensitive materials, organic semiconductors as p‐type or n‐type sensing layers have attracted great attention. Compared with conventional gas sensor materials based on metal oxide or composite systems, metal‐free organic molecules have great advantages of well‐defined structures, cost‐effectiveness, low working temperature, easy fabrication, and flexible applications,^[^ ^8^ ^]^ which are considered to be particularly promising candidates for gas‐sensitive materials.

**Scheme 1:**
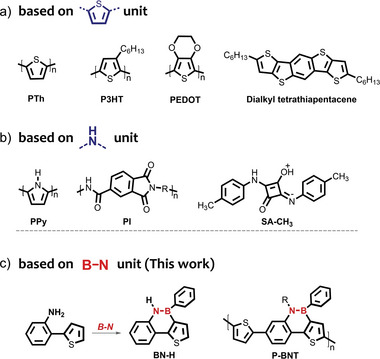
Examples of organic gas sensing materials with conventional building blocks based on a) thiophene, b) amine, and c) B‐N units (This work).

Recently, a few conjugated organic/polymer gas‐sensitive materials containing thiophene or imine units have shown excellent NH_3_ sensing performance (**Scheme** **1**) due to their various active sites.^[^ ^9^ ^]^ For example, Chi and co‐workers demonstrated a high‐performance ammonia sensor based on dialkyl tetrathiapentacene, which has ultrathin dendritic microstrips prepared via a dip‐coating method.^[^ ^10^ ^]^ Lu and co‐workers designed a squaraine of resonance‐stabilized zwitterionic structure to detect NH_3_, achieving a lower detection limit while maintaining fast response/recovery and good stability.^[^ ^11^ ^]^ In addition, sensing materials such as highly oriented PPy nanotubes,^[^ ^12^ ^]^ polythiophene (PTh) films,^[^ ^13^ ^]^ and polyimide materials^[^ ^14^ ^]^ were also employed to gain low detection limit and fast response to NH_3_. However, the accurate design of ammonia sensing materials with high response values is still a great challenge, thus limiting the development of sensors. Therefore, it is imperative to develop new organic gas‐sensitive materials with high sensitivity.

Boron nitrogen covalent bond (B‐N) is isoelectronic with carbon–carbon double bond (C═C), which can replace C═C unit in the molecular backbone, giving the molecules unique photophysical and electronic properties, such as increased photoluminescence quantum yields, tunable energy levels, and good intermolecular interactions.^[^ ^15^ ^]^ Notably, triarylboron units have empty p‐orbitals, which contribute significantly to the lowest unoccupied molecular orbital (LUMO) of the molecules and can serve as electron‐withdrawing groups, conducing to the development of stable n‐type semiconductor materials.^[^ ^16^ ^]^ In addition, the B‐N unit as an acceptor unit in the molecular structure would provide additional intermolecular dipole‐dipole interactions that would allow easy binding to the electronegative N atom, which will provide well‐defined adsorption sites.^[^ ^17^ ^]^ More importantly, the Lewis acidity of the B atom can form coordination bonds with Lewis base molecules such as ammonia, thereby modulating the electron motion state and surface charge density of active sites in molecular structures.^[^ ^18^ ^]^ Thus, the excellent structure of organic materials and the unique properties of B‐N units will provide new ideas for designing organic ammonia sensing materials with excellent performance.

Herein, a series of conjugated organic small molecules and polymers containing B‐N units were designed as metal‐free electron‐deficient organic sensing materials for the detection of ammonia. The **P‐BNT**‐based sensor can work at room temperature with an extremely high response value of 32 000 (Ra/Rg) against 40 ppm of NH_3_. Interestingly, a small‐molecule‐induced interfacial strategy was employed to modulate the recoverability of the **P‐BNT** polymer. The **BN‐H** embedded polymer system with rapid response and recovery times can still exhibit a high responsivity of 718, which is among the highest reported NH_3_ chemiresistive sensors. The sensor exhibited ultrahigh stability and selectivity. Experimental in situ Fourier transform infrared (FTIR) spectra and theoretical calculations proved that the distinctive small molecules also promoted charge transfer and enhanced electron concentration of **P‐BNT** sensing polymer, thus achieving superior B‐N‐containing material systems with excellent sensing performance. More importantly, a simple NH_3_ monitoring device based on B‐N‐based materials has been assembled, which can directly light up LED lights for practical applications without signal amplification components.

## 2 Results and Discussion

**Figure 1:**
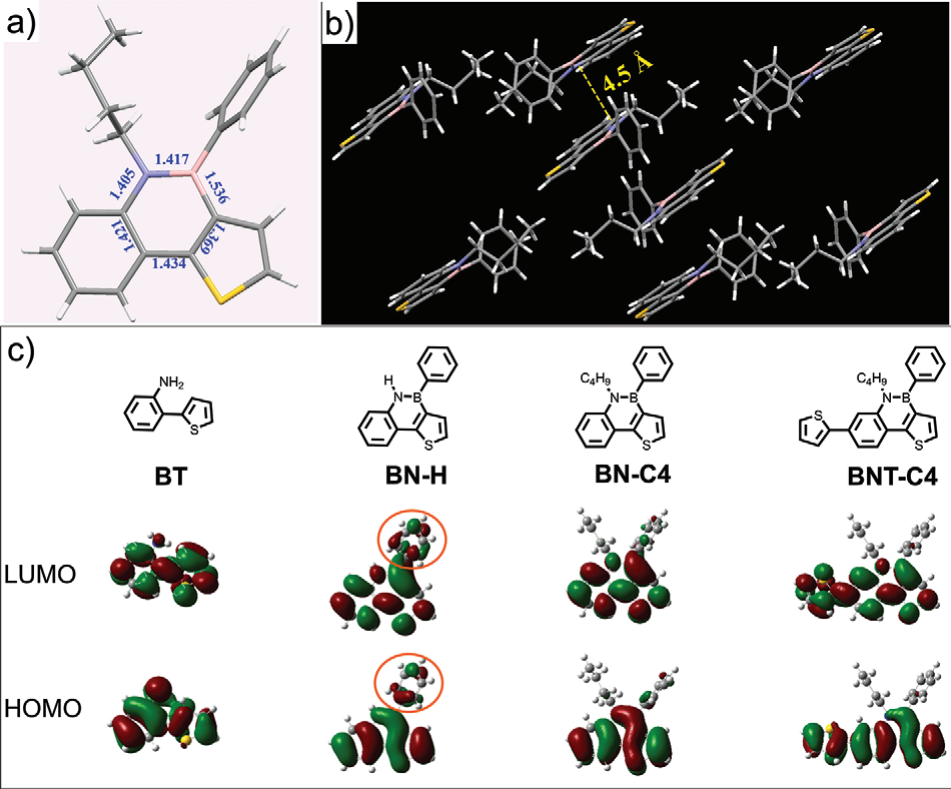
a) Single‐crystal structure and b) Stacking structure of **BN‐C4**. c) Kohn–Sham LUMOs and HOMOs of model compounds of **BT, BN‐H, BN‐C4,** and **BNT‐C4** models, based on calculations at the B3LYP/6‐31g(d,p) level.

Single crystals of **BN‐C4** were prepared by recrystallization from hexane/THF solutions (**Figure** **1**a). The X‐ray single‐crystal analysis exhibits an almost planar geometric shape of the framework (Figure S1, Supporting Information). A vertical head‐to‐tail π‐stacked orientation in the crystal packing is displayed with a mean plane distance of 4.5 Å (Figure 1b). To gain more insight into the electronic structures of **BT, BN‐H, BN‐C4**, and **BNT‐C4**, density functional theory (DFT) calculations were performed at the theoretical level of B3LYP/6‐31g(d,p) (Figure 1c). The calculation results show that the LUMO and highest occupied molecular orbital (HOMO) of all B‐N‐based organic molecules are similarly delocalized over the entire π‐conjugated frameworks (Figure S2, Supporting Information). It is noteworthy that the N‐H containing **BN‐H** shows significant charge distribution on the phenyl group, which is completely different from those of **BN‐C4** and **BNT‐C4** sensing materials with alkyl groups, indicating that the active N‐H moiety can increase charge delocalization and is more conducive to gas adsorption in such molecular systems. Moreover, the calculated C‐C bond between phenyl and thiophene units in **BN‐H** (1.4428 Å) and **BN‐C4** (1.4358 Å) is much shorter than that in **BT** (1.4720 Å) (Figure S3, Supporting Information), thus demonstrating that those C─C bonds show more double bond properties and the B‐N fused molecules are more obviously conjugated in **BN‐** **H** and **BN‐C4** than in **BT** molecule. In addition, thermal gravimetric analysis (TGA) showed that they maintained good thermal stability with a thermal decomposition temperature (*T*_d_) at 20% weight loss of 226 °C for **BN‐H** and 465 °C for **P‐BNT** (Figure S4, Supporting Information).

**Figure 2:**
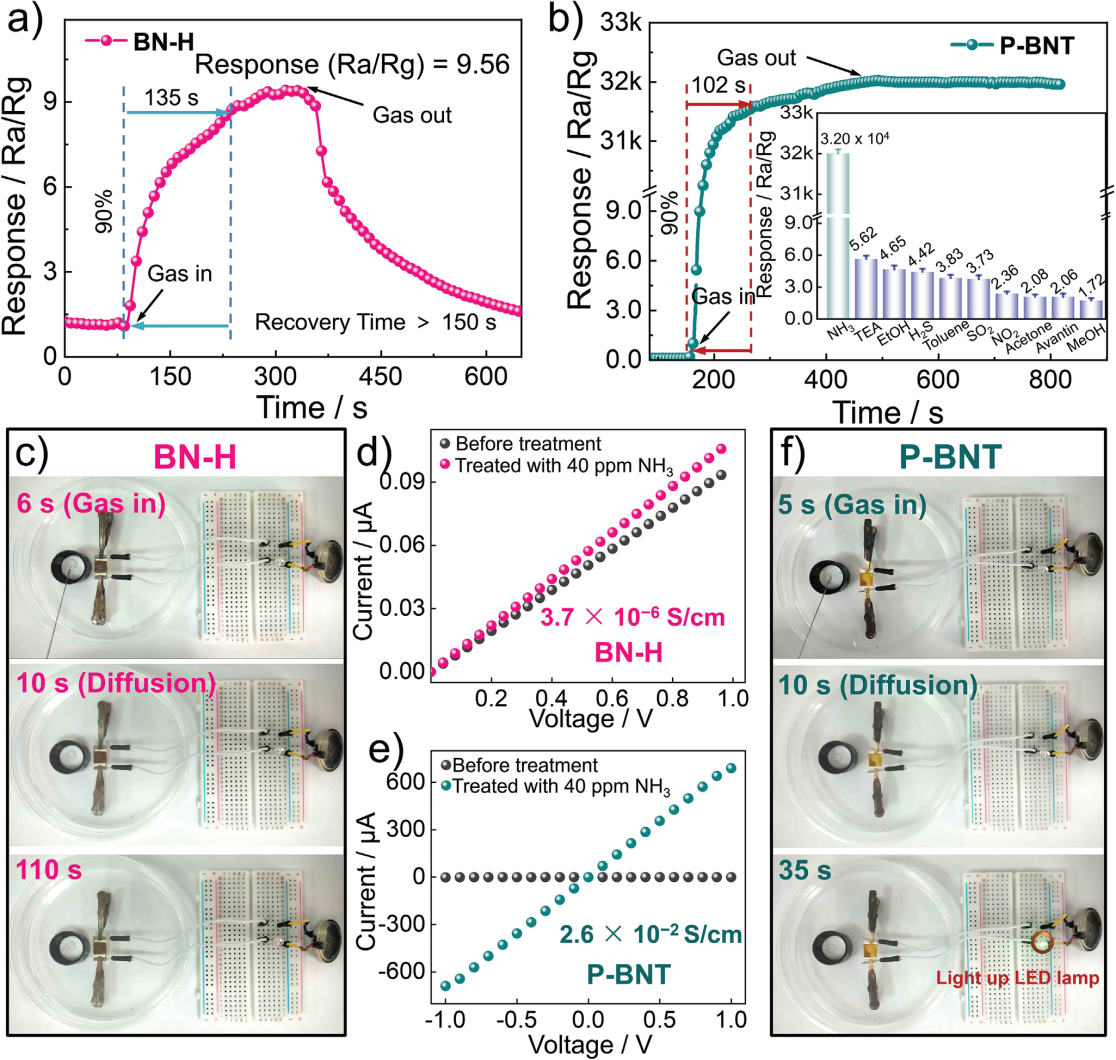
The dynamic response–recovery curves of a) **BN‐H** and b) **P‐BNT**. Inset: column chart of responses of **P‐BNT** toward different interfering gases. Photograph of the alarm system of c) **BN‐H** and f) **P‐BNT**. Current‐voltage characteristics of d) **BN‐H** and e) **P‐BNT**.(Each experiment was independently tested three times; sample size n = 3; mean ± standard deviation (mean ± SD) was analyzed using Origin software; SD reflects the degree of dispersion among individual samples; a small SD means that the value of the test is close to the average; the statistical test was two‐sided testing, the α value was 0.05 and related P values <0.0001 were analyzed by a Student's two‐side t‐test of GraphPad Prism software).

Currently, achieving high sensitivity and directional detection of NH_3_ at room temperature remains a huge challenge. In view of good semiconductor conductivity and abundant adsorption sites of the prepared B‐N‐containing sensors, simple sensing devices coating pure organic molecules were manufactured to evaluate their optimum working temperature and sensing performance (Figures S5 and S6, Supporting Information). **Figure** **2**a exhibits the typical response time (time required to increase the resistance to 90% of the saturation value) and recovery time (the time required to decrease the saturation resistance to its 10%) curves of **BN‐H** at 40 ppm of NH_3_. When exposed to NH_3_, the sensor response current shows a significant increase, exhibiting a typical n‐type semiconductor behavior. The response and recovery time values of higher than 150 s can be estimated. Good response repeatability with a low coefficient of variation (1.64%) can also be observed (Figure S7a, Supporting Information). Notably, the **P‐BNT** polymer sensor showcases an excellent responsivity over 30 000 (Ra/Rg) against 40 ppm of NH_3_, which is ≈3300 times higher than that of its B‐N organic small molecule **BN‐H** and is among the highest values of the reported ammonia sensor at room and high temperatures (Figure 2b; Table S2, Supporting Information). Unfortunately, it exhibits a prolonged recovery time because of the strong bond dissociation energy between the polymer and target analyte.

To evaluate the selectivity of the **P‐BNT** sensor, its cross‐sensitivities toward 40 ppm of various interfering gases were employed in Figure 2b inset, showing a response below 6 (Ra/Rg) toward all of these gases. The selectivity value of NH_3_ for different interfering gases (S = Response (NH_3_)/Response (gas)) varied from 5700 to 18 600, which is sufficient for the accurate detection of NH_3_ in these interfering gases. It also has good gas selectivity in **BN‐H** small molecule systems (Figure S7b, Supporting Information). The extremely high response of BN‐based sensors prompted us to assemble simple low‐energy consumption alarm systems to visually observe their response to NH_3_ (Figure 2c,f). Unlike **BN‐H** small molecule sensors (Figure 2d), the **P‐BNT**‐based sensor is illuminated in a closed device after being exposed to NH_3_ for 35 s (Movies S1 and S2, Supporting Information), which is mainly due to the significantly enhanced conductivity of **P‐BNT** (0.026 S cm^−1^) after adsorption of ammonia (Figure 2e; Figure S11, Supporting Information).

**Figure 3:**
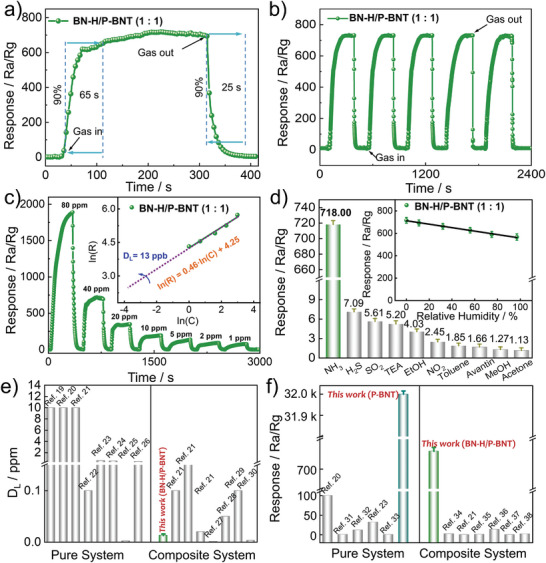
a) Dynamic response–recovery curves and b) Reproducibility of the response of **BN‐H/P‐BNT**. c) Dynamic response curves of **BN‐H/P‐BNT** toward different NH_3_ concentrations from 1 to 80 ppm, inset: the linear relationship of response versus NH_3_ concentration. d) Selectivity of the sensors to various testing gases at 40 ppm, inset: response values at different relative humidity (Each experiment was independently tested three times). e) Detection limit (D_L_) distributions and f) Response distributions of reported pure and composite organic electrochemical NH_3_ sensors and B‐N‐based sensing materials.

To further improve the response and recovery capacity of **P‐BNT**, we demonstrate a small‐molecule‐induced interfacial strategy for the suppression of strong acid‐base interaction to boost the conductivity of the sensing layer and oxidation reaction of NH_3_. Upon exposure to 40 ppm NH_3_, the **BN‐H**‐induced **P‐BNT** composite film‐based sensor **BN‐H/P‐BNT** showed a fast response and recovery time of 65 and 25 s, respectively (**Figure** **3**a; Figures S8–S9, Supporting Information), and the responsivity can reach 718, higher than the reported pure and hybrid carbon‐based chemiresistive sensors^[^ ^19^, ^20^, ^21^, ^22^, ^23^, ^24^ ^]^ (Figure 3f; Table S2, Supporting Information). The obtained sensor also exhibits an excellent response repeatability at room temperature (Figure 3b; Figure S10b, Supporting Information), which demonstrates its excellent stability. The signal curve of **BN‐H/P‐BNT** presents an excellent response‐recovery to 1–80 ppm of NH_3_ concentrations (Figure 3c). Figure 3c inset exhibits the ln–ln plots of response versus concentration of **BN‐H/P‐BNT** sensor toward NH_3_. The detection limit (D_L_) is calculated to be 13 ppb from the simulated linear equation, which is lower than most of the pure and composite systems^[^ ^25^, ^26^, ^27^, ^28^, ^29^, ^30^, ^31^, ^32^, ^33^, ^34^, ^35^, ^36^ ^]^ (Figure 3e). The cross‐sensitivities of **BN‐H/P‐BNT** toward 40 ppm of typical gases were assessed (Figure 3d). The sensor responses were <8 toward most of these gases, which revealed excellent selectivity of the metal‐free organic sensing materials. This is attributed to the electron‐deficient property of triarylboron, forming strong electrostatic interactions with NH_3_ and significantly enhancing the conductivity of **BN‐H/P‐BNT** (Figure S10a, Supporting Information). More importantly, the **BN‐H/P‐BNT** sensor can still maintain 570 (Ra/Rg) sensing response when exposed to 98% relative humidity resistance, which is attributed to the strong Lewis acid‐base interaction between ammonia and **BN‐H/P/BNT**, thus, making it suitable for real‐life and highly humid environment applications.

Gas adsorption models were established to further evaluate the interaction between B‐N‐based organic molecules and NH_3_. In these optimized structural models, B‐N (N) on organic small molecule **BN‐H** with both active N‐H and electron‐deficient B‐N units exhibit a negative charge of −0.551, higher than its NH_3_‐absorbed model, while the B‐N (N) on polymer **P‐BNT** sensor shows similar negative charges with (–0.488) and without (–0.489) NH_3_ absorption. It is worth noting that the positive charges of B‐N (B) on the **P‐BNT** model decreased by 0.015 after absorbing NH_3_. The results indicate that electronic density and electronic interaction can be regulated by constructing molecular backbones and absorbing gas molecules.^[^ ^37^ ^]^ Moreover, the well‐defined **BN‐H** and **P‐BNT** with NH_3_ exhibit dipole moments of 4.09 and 10.44 Debye (D), respectively, which are higher than those without gas molecules (1.27 D for **BN‐H** and 6.39 D for **P‐BNT**). Moreover, polymer sensors exhibit larger dipole moments than small molecule systems, indicating that polymer sensors can easily enhance local polarity and facilitate gas absorption.

**Figure 4:**
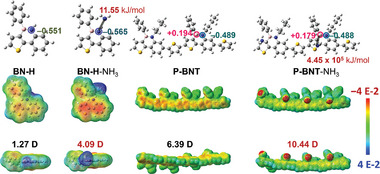
Molecular structures, electrostatic potential surface maps, adsorption energy, and molecular dipoles of **BN‐H** and **P‐BNT** with and without NH_3_ at possible binding sites.

In addition, in electrostatic potential surface (EPS) maps, the conjugated phenylthiophene unit in **BN‐H** is electronically negative after absorbing NH_3_, and the phenylthiophene unit in **P‐BNT** with NH_3_ is electronically positive, thus demonstrating that the conjugated polymer skeleton provides delocalization and transfer conditions for free electrons (**Figure** **4**). Once NH_3_ reacts with oxygen intermediates adsorbed on the surface of **P‐BNT** sensor, the electrons captured by oxygen will return to the inside of the sensor, rapidly reducing its resistance. However, such a strong adsorption energy of 445 000 kJ mol^−1^ for **P‐BNT** makes it difficult to desorb from the surface of sensing layer or react with oxygen intermediates, preventing the release of electrons. This phenomenon also explains the reason for its poor recoverability (Figure 2b).

**Figure 5:**
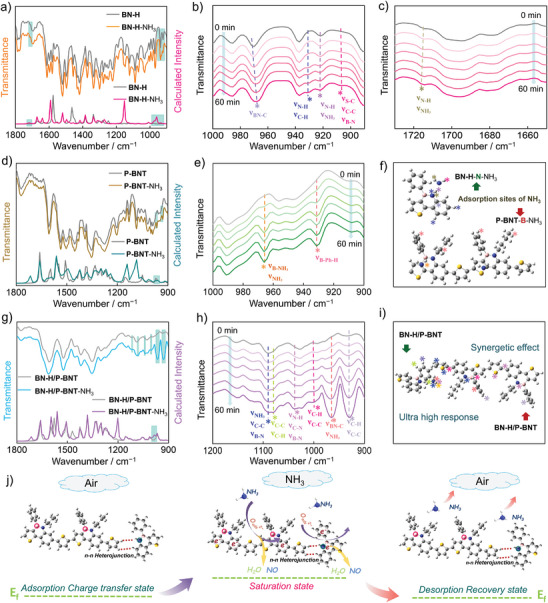
Calculated FTIR spectra of a) **BN‐H**, d) **P‐BNT,** and g) **BN‐H/P‐BNT** before and after the adsorption of NH_3_. In situ FTIR spectra of b‐c) **BN‐H**, e) **P‐BNT,** and h) **BN‐H/P‐BNT** after NH_3_ adsorption. Possible NH_3_ adsorption sites on f) **BN‐H** and **P‐BNT**, and i) **BN‐H/P‐BNT**. j) Schematic illustration of NH_3_ sensing mechanism of **BN‐H/P‐BNT**.

To elucidate the interaction sites of these organic molecules in the sensing process, gas sensing mechanisms were investigated by in situ FTIR spectra and DFT calculation. Possible theoretical models (N‐H‐NH_3_, C‐B‐NH_3_, C‐N‐NH_3_, and N‐B‐NH_3_) were constructed to definite the adsorption sites of NH_3_ at these predicted locations. Combined with in situ and calculated FTIR spectra, the N‐H on **BN‐H** and B‐N on **P‐BNT** corresponded well with the measured data, indicating the different active sites caused by electron‐deficient B‐N units and active N‐H (**Figure** **5**a,d,g). As the NH_3_ gradually increased, various distinct bands were found at 900–1820 cm^−1^ for **BN‐H**, **P‐BNT**, and **BN‐H/P‐BNT**. Notably, peaks at corresponding positions appeared in calculated FTIR spectra for aromatic backbones and ammonia molecules during the sensing process, which is attributed to the B‐N, N‐H, and C‐C/C‐N vibration signals. It is noteworthy that the peaks at 900–940 cm^−1^ for **BN‐H** are generated by the adsorption of NH_3_ on N‐H part (Figure 5a–c). While for **P‐BNT**, the peaks at 910 and 970 cm^−1^ greatly increase in the gradually increasing ammonia atmosphere (Figure 5d,e), attributing to the absorption of NH_3_ by electron‐deficient triarylboron (Figure 5f). Interestingly, in the **BN‐H/P‐BNT** composite, the characteristic peaks exhibit multiple variations at 900–1100 cm^−1^ (Figure 5h), and the peak intensity changes significantly compared to those of pure **BN‐H** and **P‐BNT**, which is also supported by the calculated FTIR spectra (Figure 5g). In addition, the FTIR data of the diversified ammonia vibration peaks at 960, 1060, and 1080 cm^−1^ also demonstrated the successful synergistic effect between **BN‐H** and **P‐BNT**, proving its excellent sensing performance (Figure 5i). As NH_3_ can donate electrons to **P‐BNT** through B‐N active sites, thereby increasing electron concentrations, optimizing conductivity, and improving sensing performance. Importantly, the N‐H fractions in small molecule **BN‐H** promoted charge distribution on phenyl groups, and more easily narrowed the electron depletion layer and accumulation layer. Meanwhile, the formation of heterostructures between **BN‐H** and **P‐BNT** can reduce the potential barrier energy in NH_3_ and promote charge transfer between NH_3_ and the sensing layer, which facilitates NH_3_ response and recovery time.^[^ ^38^ ^]^ The sensing mechanism of NH_3_ can be described by Wolkentein's model (Figure S12, Supporting Information) and the response process is as follows:^[^ ^39^ ^]^

(1) $$O_{2}\left(g\right)\to O_{2}\left(\mathrm{ads}\right)$$

(2)

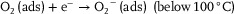

(3) $$4NH_{3}+5{O_{2}}^{-}\to 4\mathrm{NO}+6H_{2}O+5e^{-}$$

Moreover, the **BN‐H/P‐BNT** with fibrous and needle‐shaped morphology also corresponds well with its excellent sensing activity (Figure S13, Supporting Information), which effectively regulates the motion state and pathway of electrons in thus sensing materials, thereby changing the resistivity. These distinctive small molecules also promoted charge transfer and enhanced the electron concentration of the **P‐BNT** sensing polymer.

## 3 Conclusion

In summary, novel B‐N‐based organic molecules were designed for ammonia detection. The as‐fabricated sensing device based on **P‐BNT** exhibited the highest sensitivity of 32 000 against 40 ppm of NH_3_ at room temperature. To weaken the bond dissociation energy between **P‐BNT** and NH_3_ caused by strong acid‐base interaction, a small‐molecule‐induced interfacial strategy is employed to optimize the response and recovery time. As a result, the **BN‐H/P‐BNT** composite sensor with well‐modulated surface charge density and electron motion state still endows an extremely high response. Importantly, its recovery time unprecedentedly improved to 25 s, accompanied by excellent stability, selectivity, and relative humidity resistance. Experimental and theoretical calculation results demonstrated that B‐N covalent bonds can produce abundant active sites and efficient absorption for gas sensing. In situ FTIR spectra further proved that the electron‐deficient B‐N units with strong Lewis acid are principal for excellent performance. This work provides a promising way for developing efficient organic sensors via the B‐N construction strategy.

## Conflict of Interest

The authors declare no conflict of interest.

## Supporting information

Supporting Information

Supplemental Movie 1

Supplemental Movie 2

## Data Availability

The data that support the findings of this study are available in the supplementary material of this article.
