# Supplementary material for: High‐Performance Room Temperature Ammonia Sensors Based on Pure Organic Molecules Featuring B‐N Covalent Bond
Source: Adv Sci (Weinh). 2024 Mar 14;11(19):2308483. doi: 10.1002/advs.202308483 (PMC11109643; doi:10.1002/advs.202308483)
Supplement: Supplementary file 1 — Supporting Information [file ADVS-11-2308483-s002.pdf]

ADVANCED SCIENCE
Open Access

Supporting Information

for *Adv. Sci.*, DOI 10.1002/advs.202308483

High-Performance Room Temperature Ammonia Sensors Based on Pure Organic Molecules Featuring B-N Covalent Bond

Qian Wang, Meilong Wang, Kunpeng Zheng, Wanneng Ye, Sheng Zhang, Binbin Wang and Xiaojing Long**

High-Performance Room Temperature Ammonia Sensors Based on Pure Organic Molecules Featuring B-N Covalent Bond

Qian Wang[a],+, Meilong Wang[a],+, Kunpeng Zheng[a], Wanneng Ye[a], Sheng Zhang[b], Binbin Wang[a], Xiaojing Long*[a]*

Q. Wang, M. Wang, K. Zheng, Prof. W. Ye, Prof. S. Zhang, B. Wang, Prof. X. Long

[a] State Key Laboratory of Bio-fibers and Eco-textiles, Collaborative Innovation Center of Shandong Marine Biobased Fibers and Ecological Textiles, Institute of Marine Biobased Materials, College of Materials Science and Engineering, Qingdao University, Qingdao 266071, P. R. China

E-mail: ywn@qdu.edu.cn; longxj@qdu.edu.cn.

[b] Institute of Nanoscience and Engineering, Henan University, Kaifeng 475004, P. R. China

Content

1. Experimental details.. S3

2. Structural characterization.. S8

3. DFT calculations.. S9

4. Thermogravimetric analysis S10

5. Gas sensing testing.. S11

6. Morphology S16

7. NMR spectra.. S17

1. Experimental details

General. ^1H NMR spectra were measured with a Bruker AV-400 spectrometer in $CDCl_3$ at 25 °C. Chemical shifts are reported in ppm using $CHCl_3$ (7.26 ppm) for ^1H NMR, using $CDCl_3$ (77.16 ppm) for ^{13}C NMR, and using $CDCl_3$ for ^{11}B NMR as an internal standard. Thermal analysis was performed on a TG 209 instrument under nitrogen flow at a heating rate of 10 °C min^{-1}. The morphologies and structures of the samples were characterized by using a field emission scanning electron microscope (SEM, Quanta 250 FEG) and transmission electron microscopy (TEM) (JEM-1011; JEOL Co., Japan) operated at an accelerating voltage of 100 kV. The current (I)-voltage (V) curves were measured by a source Meter (2612B, Keithley) at room temperature. Fourier transform infrared spectroscopy (FTIR) spectra were recorded between 4000 and 650 cm^{-1} using a PerkinElmer Spectrum 100 FTIR spectrometer. And exposing 40 ppm ammonia gas in a vacuum environment at room temperature.

Gas sensing measurements. Sensing device fabrication and gas-sensing performance measurements: **BN-H** (3.0mg), **P-BNT** (3.0mg), or **BN-H/P-BNT** (3.0mg) and dichloromethane were added to a mortar and ground for 5 minutes. Next, 10 μL solution was coated on interdigital electrodes (IDEs) to form uniform sensing films. After drying, the sensor element was aged overnight at 200 °C to improve stability. Gas sensing measurements were carried out by employing a Keithley electrometer (2611B) integrated with a customized gas testing chamber at an input signal of 3 V. The static liquid–gas distribution method was employed to calculate the volume (μL) toward the desired concentration (ppm) of test analytes. The concentration of test analytes (ammonia, acetone, triethylamine, methanol, nitrogen dioxide, and ethanol) was measured using Eq. (1) and injected into the evaporator inside the chamber.

$$C_{ppm} = \frac{\delta \times V_r \times R \times T}{M \times P_b \times V_b} \times 10^6 \text{................ Eq. (1)}$$

where δ is the density of the analyte, Vr is the volume of analyte injected, R is the universal gas constant, T is the absolute temperature, M is the molecular weight, P_b is the pressure inside the chamber and V_b is the volume of the chamber. The required volume of test analytes was taken using a microliter syringe. The current of the sensor was continuously monitored until it reached the steady state in the presence of a desired concentration of test analytes. Once the steady state was reached,

the chamber was exposed to the ambient atmosphere to ensure the reversibility of the sensor, and the response characteristics of the sensor were monitored continuously. The testing device and process are shown in the following figure:

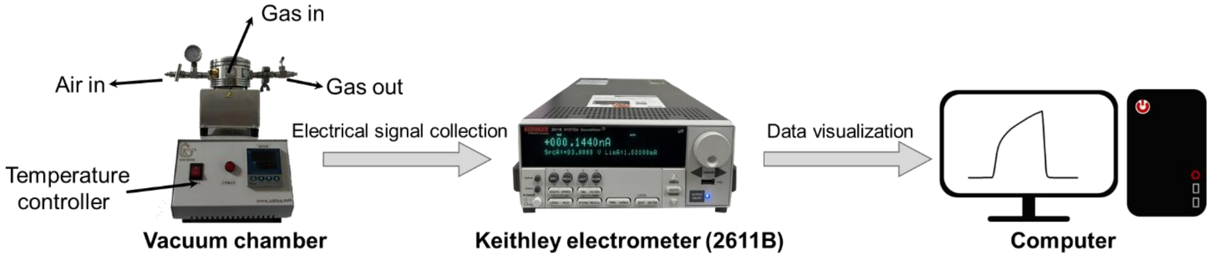

The response of the gas sensor (S) is defined as the ratio of resistance (Ra) of the sensor in the air to resistance (Rg) of the sensor after treatment with measured gas, that is, S = Ra/Rg. According to the least-squares method of fitting in the linear regime, the theoretical detection limit (D_L) of the gas sensor is the value of gas concentration when the sensor response is three times greater than the standard deviation of the noise signal (RMS_{noise}), which can be derived as follow:

$$D_{\mathrm{L}}(ppm) = \frac{3 \cdot rms_{noise}}{slope} = \frac{3}{slope} \cdot \sqrt{\frac{V_{x^2}}{N}}$$

where N is the number of data points used in plot fitting, V_{x^2} is standard deviation of the data points used, and the slope is the fitting plot of response versus ammonia concentration.

Synthesis

All starting materials and solvents, unless otherwise noted, were obtained from Shanghai Aladdin Biochemical Technology Co., Ltd. All reactions were performed under an argon atmosphere. Organic solvents including CH_2Cl_2, THF, and toluene were dried before use. Other solvents and reagents were used without further purification unless otherwise mentioned.

(1) Synthesis of **BN-H**:

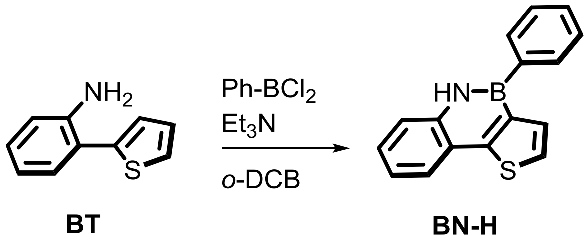

BN-H: BT (210.3 mg, 1.20 mmol) were placed in a two-necked flask under argon, and then dried

o-DCB (6 mL) was added. Ph-BCl_2 (381.2 mg, 2.40 mmol), Et_3N (727.4 mg, 7.19 mmol) were added drop by drop. After the mixture was stirred at 180 °C for 24 h, After removing the solvents at reduced pressure, it was purified by silica gel column chromatography (CH_2Cl_2/hexane = 1/1). **BN-H** was obtained as a white powder in 90% yield (281.6 mg, 1.08 mmol). ^{1}H NMR (400 MHz, $CDCl_3$, 25 °C): δ 8.03 (m, 1H), 7.96–7.76 (m, 4H), 7.56–7.36 (m, 7H), 7.26 (s, 2H). ^{13}C NMR (100 MHz, $CDCl_3$) δ 139.2, 152.1, 137.9, 133.1, 132.1, 131.6, 129.3, 128.3, 127.7, 125.9, 124.2, 123.6, 121.8, 11.4, 118.9, 116.7; ^{11}B NMR (128 MHz, $CDCl_3$) δ 33.67.

(2) Synthesis of **BN-C4**:

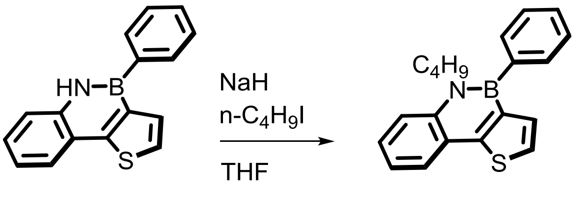

BN-C4: **BN-H** (148.9 mg, 0.57 mmol), and NaH (34.5mg, 1.43 mmol) were placed in a two-necked flask under argon, and then dried THF (7 mL) was added. After the mixture was stirred at 70 °C for 2 h, I-C_4H_9 (115.4 mg, 0.63 mmol) was added under argon. The mixture continues to be stirred for 12 h at 70 °C. After removing the solvents at reduced pressure, the yellow oil was purified by silica gel column chromatography (CH_2Cl_2/hexane = 1/8). **BN-C4** was obtained as a creamy white powder in 85% yield (153.7 mg, 0.48 mmol). ^{1}H NMR (400 MHz, $CDCl_3$, 25 °C): δ 8.08 (m, 1H), 7.64 (m, 1H), 7.60–7.38 (m, 6H), 7.32–7.27 (m, 2H), 7.19 (m, 1H), 4.18–4.10 (m, 2H), 1.80–1.69 (m, 2H), 1.27 (m, 2H), 0.82 (m, 3H). ^{13}C NMR (100 MHz, $CDCl_3$) δ 150.9, 138.9, 132.6, 132.1, 127.8, 127.7, 127.5, 125.9, 125.8, 123.6, 123.3, 121.4, 116.7, 116.6, 53.4, 47.9, 32.4, 32.3, 20.2, 13.7; ^{11}B NMR (128 MHz, $CDCl_3$) δ 36.35.

(3) Synthesis of **BNT-C4**:

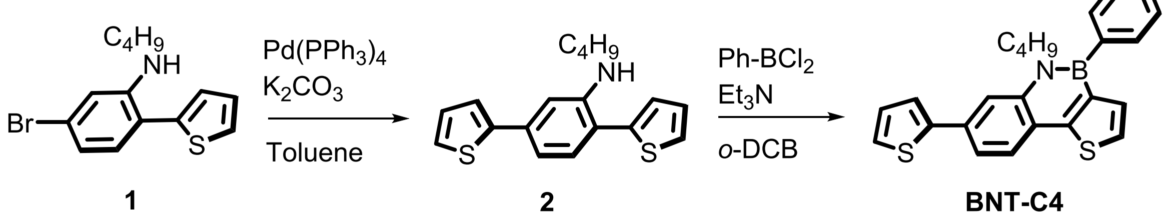

BNT-C4: The compound 1 (148.9 mg, 0.48 mmol), Thiophene-2-boronic acid pinacol ester (115.2

mg, 1.10 mmol), $Pd(PPh_3)_4$ (27.9 mg, 0.024 mmol) and K_2CO_3 (1.20 mL, 2.41 mmol, 2M) were placed in a two-necked flask under argon, and then dried toluene (8 mL) was added. After being stirred at 80 °C for 12 h, the solvents were removed and the orange solid was purified by silica gel column chromatography (CH_2Cl_2/hexane = 1/5). 3 was obtained in 91% yield (137.8 mg, 0.44 mmol). Furthermore, under argon, the compound 2 (131.7 mg, 0.42 mmol), Ph-BCl_2 (131.8 mg, 0.83 mmol), Et_3N (282.3 mg, 2.79 mmol) were placed in a two-necked flask under argon, and then dried *o*-DCB (6 mL) was added. After the mixture was stirred at 180 °C for 24 h, After removing the solvents at reduced pressure, it was purified by silica gel column chromatography (CH_2Cl_2/hexane = 1/5). **BNT-C4** was obtained as a Light brown powder in 86% yield (142.5 mg, 0.36 mmol). ^{1}H NMR (400 MHz, $CDCl_3$, 25 °C): δ 8.06 (m, 1H), 7.86 (s, 1H), 7.57 (m, 3H), 7.52–7.38 (m, 4H), 7.37–7.28 (m, 2H), 7.23–7.12 (m, 2H), 4.22–4.14 (m, 2H), 1.87–1.75 (m, 2H), 1.40–1.23 (m, 2H), 0.89 (m, 3H). ^{13}C NMR (100 MHz, $CDCl_3$) δ 150.6, 144.8, 139.2, 133.7, 132.8, 132.6, 132.2, 132.1, 128.3, 127.7, 127.6, 126.4, 125.2, 123.7, 123.8, 122.6, 47.9, 32.3, 20.2, 13.8; ^{11}B NMR (128 MHz, $CDCl_3$) δ 36.47.

(4) Synthesis of **P-BNT**:

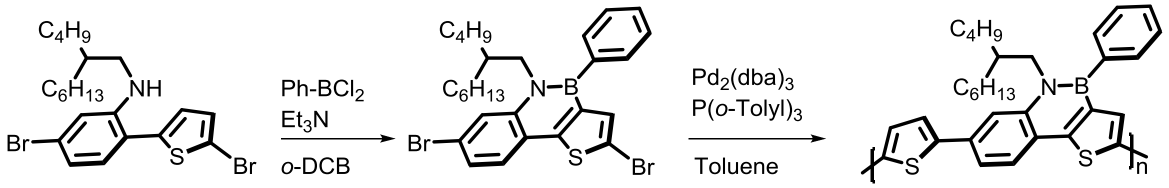

3 4 P-BNT

P-BNT: The compound 3 (170.5 mg, 0.34 mmol), Ph-BCl_2 (107.7 mg, 0.68 mmol), Et_3N (205.4 mg, 2.03 mmol) were placed in a two-necked flask under argon, and then dried *o*-DCB (5 mL) was added. After the mixture is stirred at 180 °C for 24 h. After removing the solvents under reduced pressure, it was purified by silica gel column chromatography (hexane). The compound 4 was obtained as a white powder in 92% yield (183.2 mg, 0.31 mmol). Furthermore, under argon, the compound 4 (146.8 mg, 0.25 mmol), 2,5-bis(triMethylstannyl)thiophene (102.4 mg, 0.25 mmol), $Pd_2(dba)_3$ (4.6 mg, 0.005 mmol) and P(*o*-Tolyl)$_3$ (12.2 mg, 0.04 mmol) were placed in a two-necked flask under argon and then dried toluene (5 mL) was added. After being stirred at 120 °C for 48 h, the mixture was extracted with CH_2Cl_2 (100 mL) and water, and the organic phase was dried over anhydrous

Na_2SO_4. The end-capping reaction was carried out by adding bromobenzene (78.1 mg, 0.50 mmol). After the solvents were removed, the residue was dispersed in ethanol and the precipitate was collected. The polymer was purified by Soxhlet extraction with acetone, hexane, and THF to remove impurities and catalysts as well as the residual monomers. The **P-BNT** was obtained as a yellow-green powder in 78% yield (196.3 mg,). ^{1}H NMR (400 MHz, $CDCl_3$, 25 °C): δ 8.34–7.97 (m, 1H), 7.95–7.30 (m, 7H), 7.23–6.64 (m, 3H), 4.49–3.81 (s, 2H), 2.05–1.69 (s, 2H), 1.44–0.66 (m, 21H). ^{11}B NMR (128 MHz, $CDCl_3$): δ 32.5.

Statistical Analysis

1. Pre-processing of data: TG, *in situ* FTIR, I-V and ammonia sensing data were converted into TXT format by the corresponding instruments, and plotted by Origin software without normalization and evaluation of outliers. The data of SEM, TEM, and DFT calculations were in the form of pictures or numbers, which were directly drawn by PowerPoint software without conversion, normalization, and evaluation of outliers.

2. Data presentation: The electrochemical measurements were independently tested three times to avoid any incidental error. The related error bars (presented in the form of mean ± SD) were also shown in the manuscript and supporting information. The sample size for each statistical analysis: The sample size of the related electrochemical measurements was three. Statistical methods used to assess significant differences with sufficient details: The statistical test was two-sided testing, the α value was 0.05, and related P values were analyzed by Student's two-sided t-test and showed in the Manuscript and Supporting Information.

3. Software used for statistical analysis: The related software were Origin, Powerpoint, and GraphPad Prism.

2. Structural characterization

Single crystal X-ray diffraction measurements were performed by Rigaku SmartLab X-ray diffractometer with Cu Kα radiation (λ = 1.5418 Å). The structures were solved by the direct method (SHELXL-97) and refined by the full-matrix least-squares on F2 (SHELXL-97). CCDC 2304776 (**BN-C4**) contains the supplementary crystallographic data for this paper. The data can be obtained free of charge from The Cambridge Crystallographic Data Centre via www.ccdc.cam.ac.uk / data request /cif.

Single crystals of BN-C4 suitable for X-ray crystallographic analysis were obtained by recrystallization from Hexane/THF solutions. Crystal data of **BN-C4**: $C_{20}H_{20}BNS$; FW =317.24, Monoclinic, space group C 2/c, a = 24.6771(8) Å, b = 9.7027(3) Å, c = 18.2274(7) Å, α = 90°, β = 125.748 (5)°, γ = 90°.

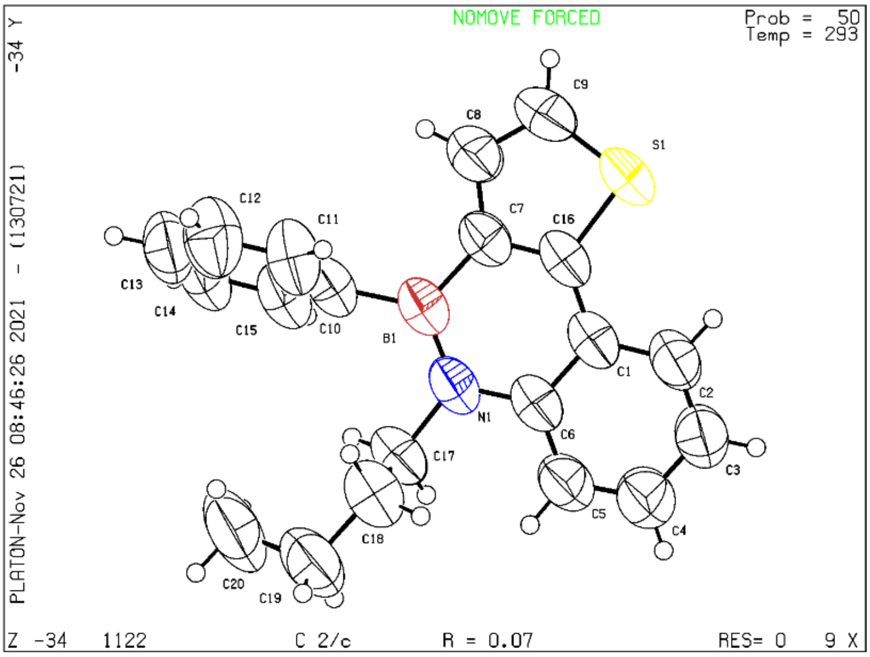

Figure S1. ORTEP diagrams for the molecular structure of **BN-C4**. Thermal ellipsoids are drawn at the 50% probability level. Hydrogen atoms have been omitted for the sake of clarity.

3. DFT calculations

The structural optimizations and DFT calculations of the model compounds **BT, BN-H, BN-C4,** and **BNT-C4** were performed using Gaussian 09 program at the B3LYP/6-31G(d,p) level of theory[1].

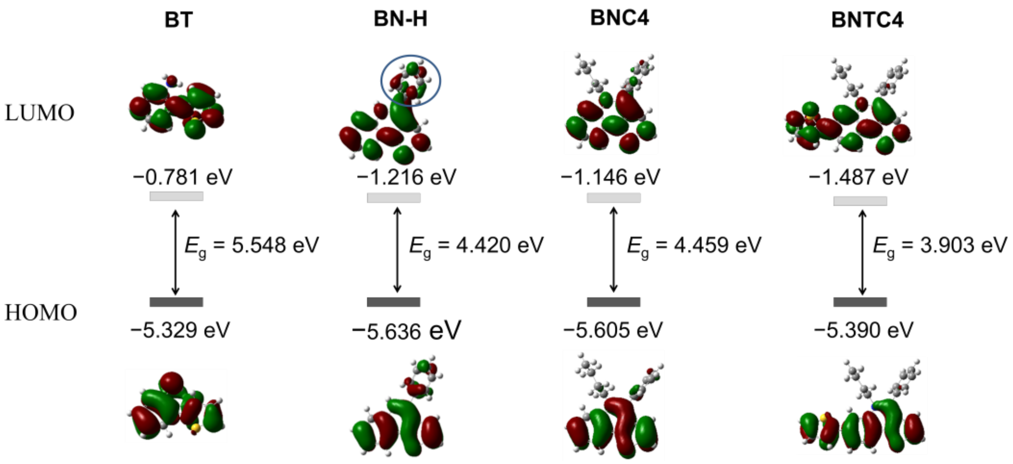

Figure S2. Kohn–Sham LUMO/HOMO and energy levels of **BT, BN-H, BN-C4,** and **BNT-C4**.

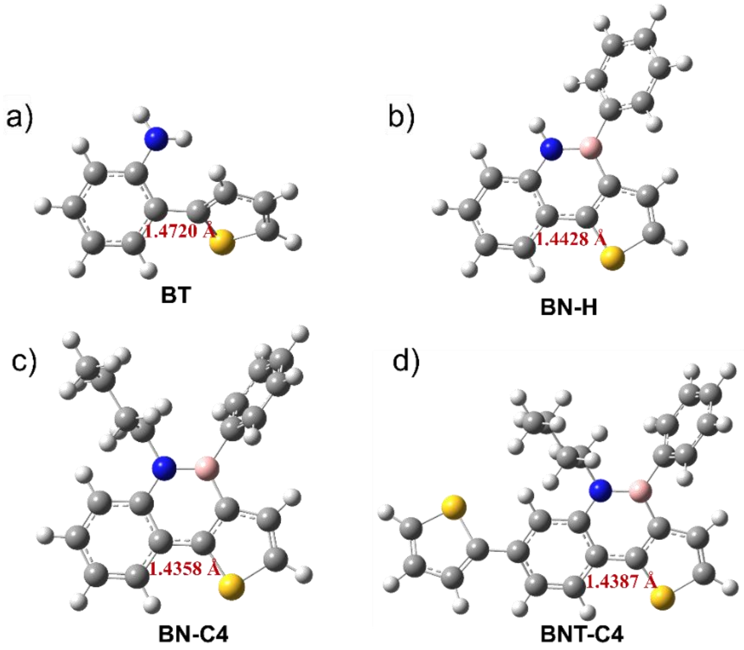

Figure S3. The calculated C-C bond of model compounds of **BT**, **BN-H**, **BN-C4**, and **BNT-C4**

based on calculations at the B3LYP/6-31g(d,p) level.

4. Thermogravimetric analysis

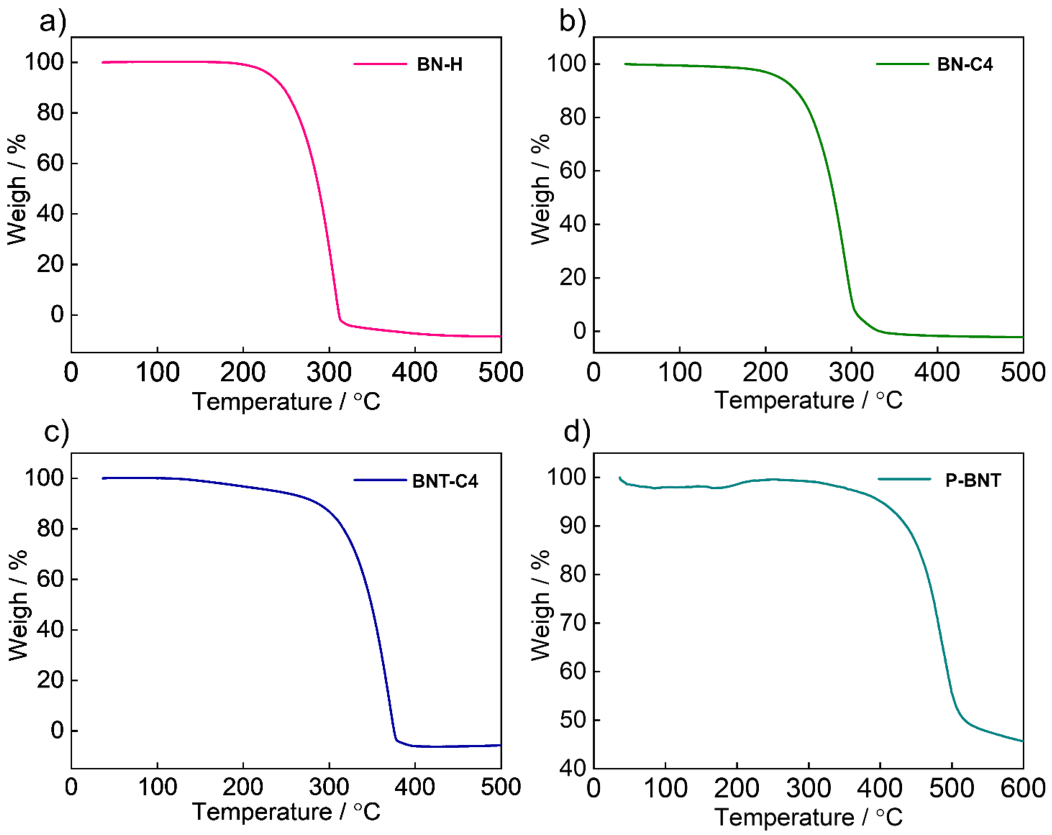

Figure S4. The thermal properties of **BN-H**, **BN-C4**, **BNT-C4,** and **P-BNT**. They show good thermal stability with thermal decomposition temperature (T_d) at 20% weight loss of 226 °C for **BN-H,** 253 °C for **BN-C4,** 316 °C for **BNT-C4,** and 465 °C for **P-BNT**.

5. Gas sensing testing

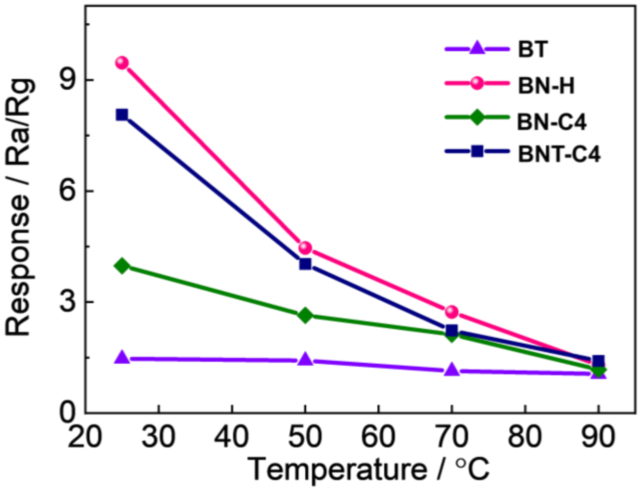

Figure S5. Response of **BT, BN-H**, **BN-C4**, and **BNT-C4** at different operating temperatures (i.e., 25, 50, 70, and 90 °C). The optimal operating temperature of the sensors is room temperature, which can meet daily life applications.

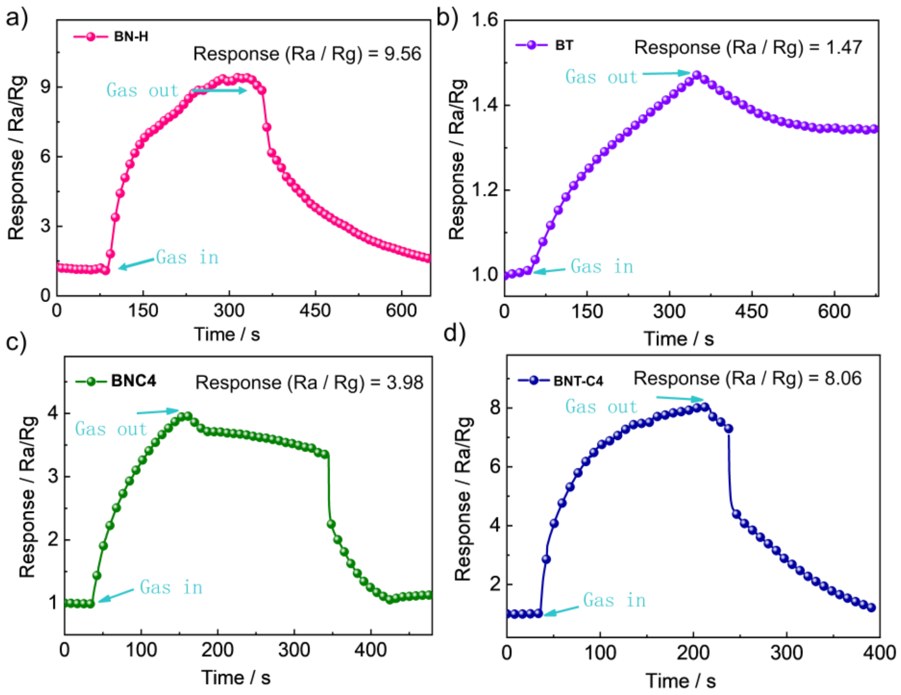

Figure S6. a-d) Dynamic response–recovery curves of **BN-H**, **BT**, **BN-C4**, and **BNT-C4** to 40 ppm of NH_3 at 25 °C. **BN-H** sensor to 40 ppm of NH_3 has the highest response value (9.56) among the four small molecules at room temperature.

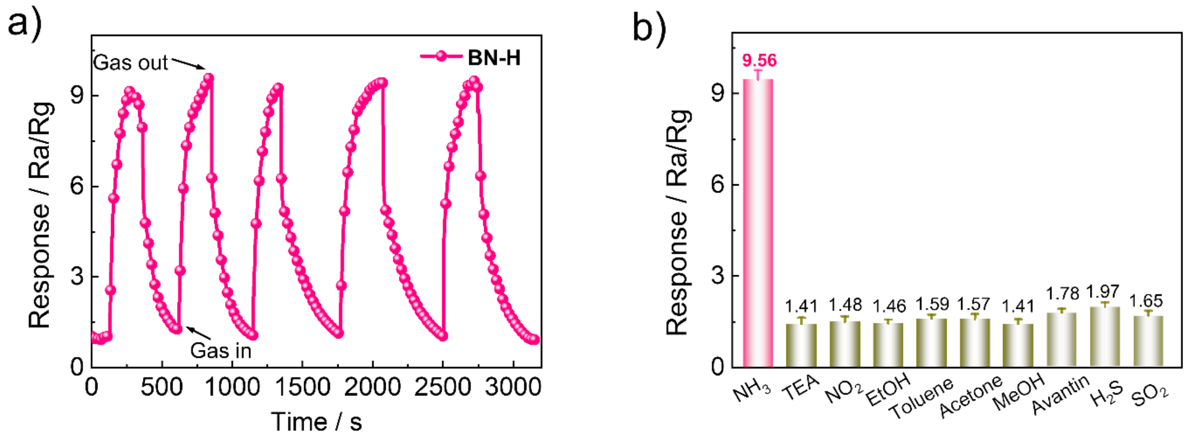

Figure S7. a) Reproducibility of the response of **BN-H** to 40 ppm NH_3 at room temperature. b) column chart of responses of **BN-H** toward different interfering gases at 40 ppm at room temperature (Each experiment was independently tested three times).

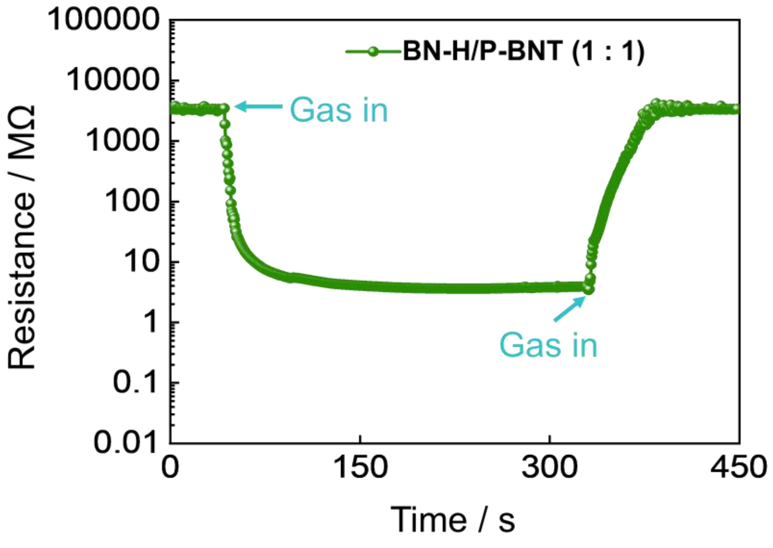

Figure S8. The resistance response/recovery curve of the **BN-H/P-BNT** (1:1)-based sensor to 40 ppm of NH_3.

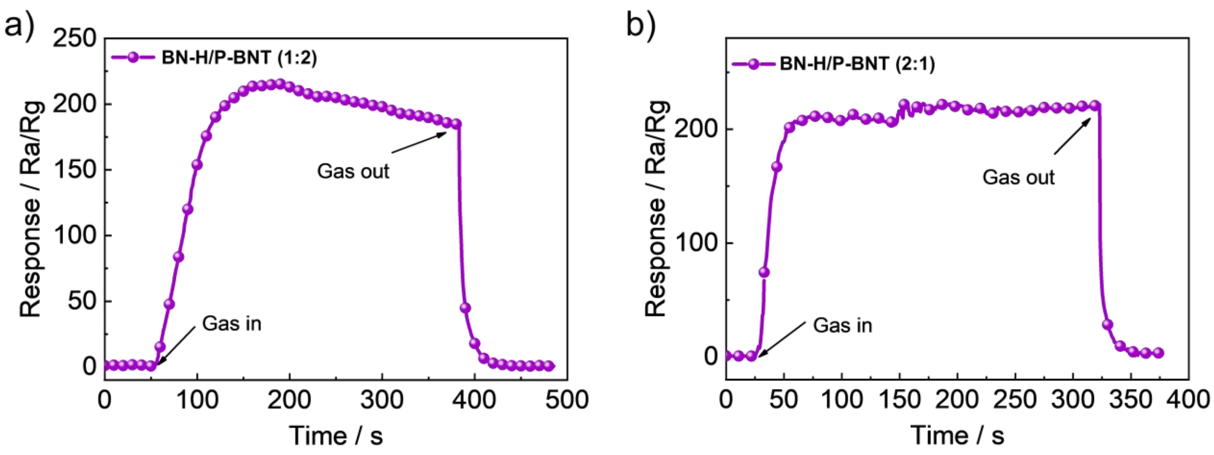

Figure S9. Dynamic response–recovery curves of a) **BN-H/P-BNT** (1:2) and b) **BN-H/P-BNT** (2:1) to 40 ppm of NH_3 at room temperature. The composite systems **B-NH/P-BNT** (1:1)-based sensor showed excellent sensing performance.

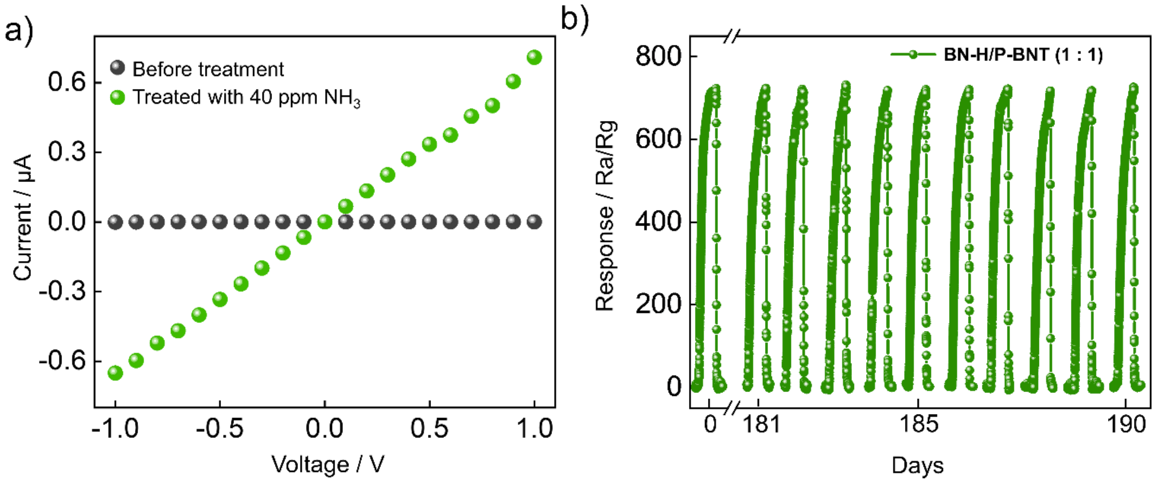

Figure S10. a) Current-voltage characteristics of **BN-H/P-BNT** (1:1). The conductivity of triarylboron system increased significantly under the NH_3 atmosphere. b) The long-term stability testing of **BN-H/P-BNT** (1:1)-based sensors.

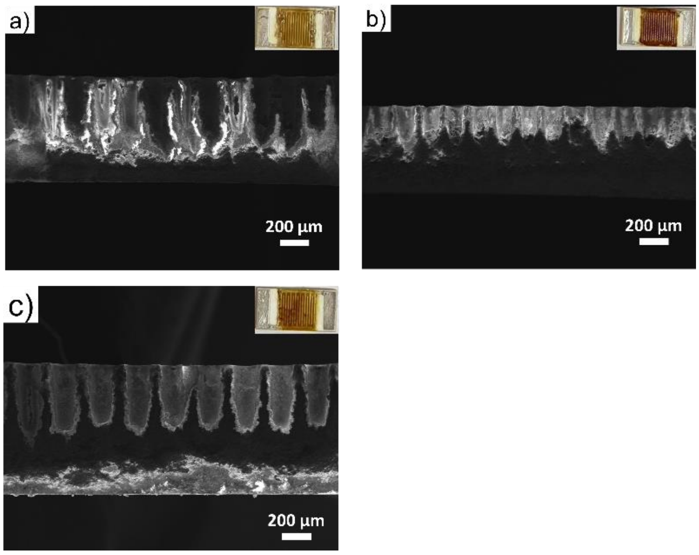

Figure S11. Electrode morphology of a) **BN-H**, b) **P-BNT**, and c) **BN-H/P-BNT** (Inset: The photography of devices). The film thickness of 294 μm for **BN-H**, 268μm for **P-BNT** and 273 μm for **BN-H/P-BNT**, which can be used to calculate the conductivity. Conductivity (σ) is calculated by using the equation: $\sigma = (I / V) \times L / (w \times d)$, where I is the current, V is the voltage, d is the thickness of the film, L and w are the length and width of the electrode.

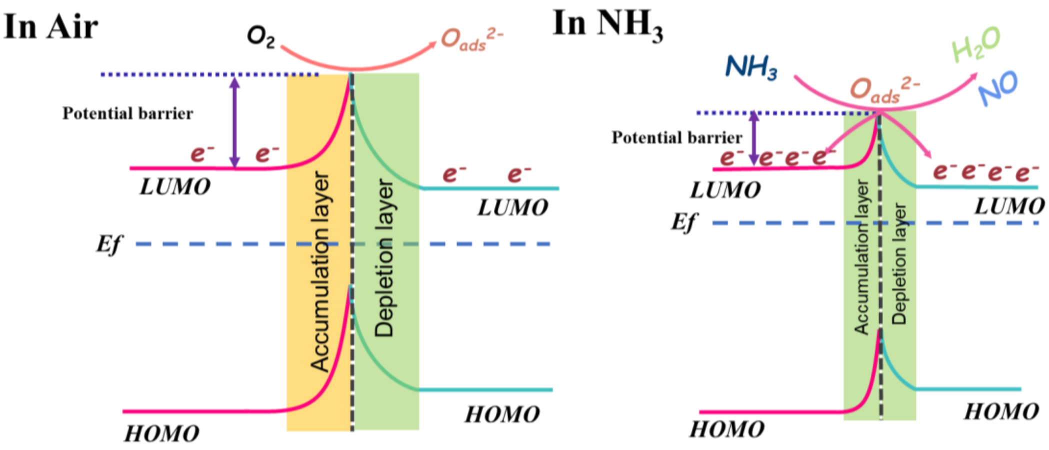

Figure S12. The sensing mechanism of **BN-H/P-BNT**-based sensor to NH_3.

Table S1. Summary of the response and recovery times at different concentrations.

	80 ppm	40 ppm	20 ppm	10 ppm	5 ppm	2 ppm	1 ppm
Response Time	179s	65 s	57 s	55 s	50 s	47 s	45 s
Recovery Time	38 s	25 s	28 s	23 s	27 s	29 s	26 s

Table S2. Summary of benchmark test results for organic ammonia sensors.

Sensing materials	Response (Ra/Rg)	Response/recovery time	Lowest detection limit	References
Dialkyl tetrathiapentacene	100 (50 ppm)	36 s/10 s	10 ppm	2
Naphthalene diimide derivative	N.A.	5 s/20 s	10 ppm	3
Monolayer pentacene	60% (40 ppm)	N.A.	10 ppm	4
Pentacene thin film	N.A.	500 s/N.A.	0.5 ppm	5
Perylene diimide	12 (40 ppm)	28 s/40 s	0.56 ppm	6
P3HT	N.A.	N.A.	10 ppm	7
Squaraine thin film	32 (64 ppm)	44 s/135 s	40 ppb	8
PQT-12	56.4% (80 ppm)	45 s/85 s	404 ppb	9
**P-BNT**	320000 (40 ppm)	40 s/N.A.	N.A.	This work
**BN-H**	9.56 (40 ppm)	135 s/＞150 s	198 ppb	This work
**BN-H/P-BNT**	718(40 ppm)	65 s/25 s	13 ppb	This work

6. Morphology

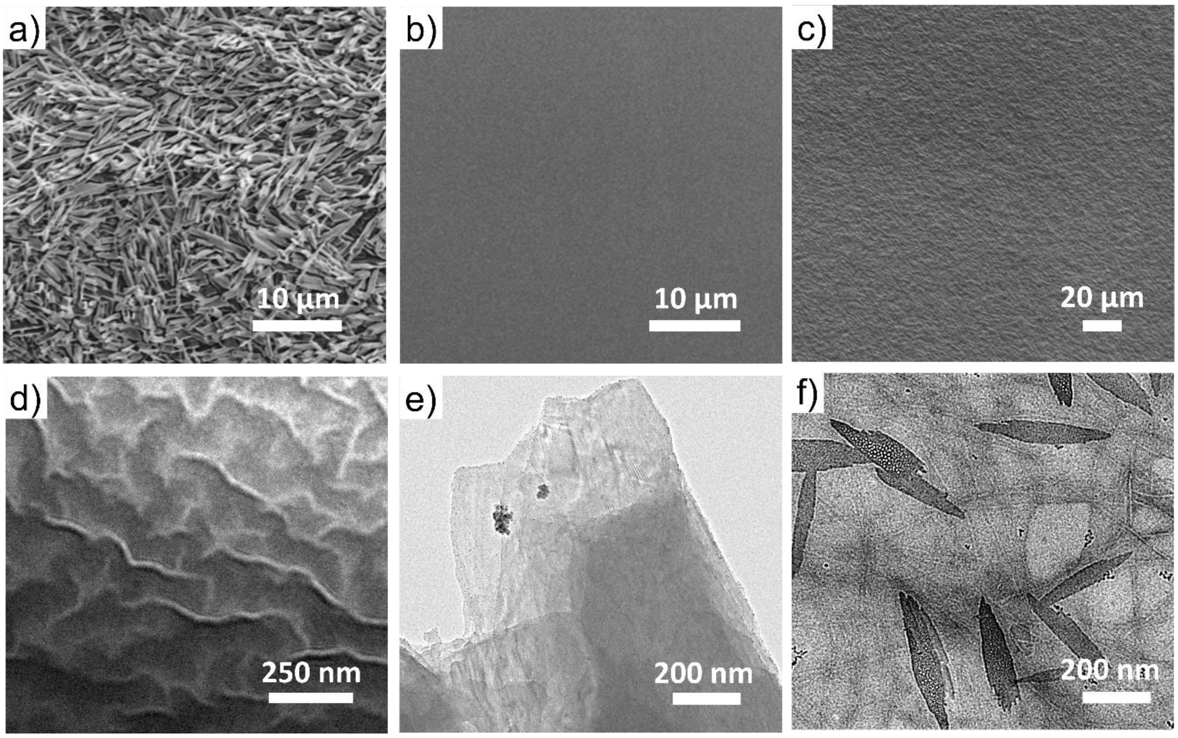

Figure S13. SEM images of a) **BN-H**, b) **P-BNT**, and c) **BN-H/P-BNT** (1:1). TEM images of d) **BN-H**, e) **P-BNT**, and f) **BN-H/P-BNT** (1:1).

7. NMR spectra

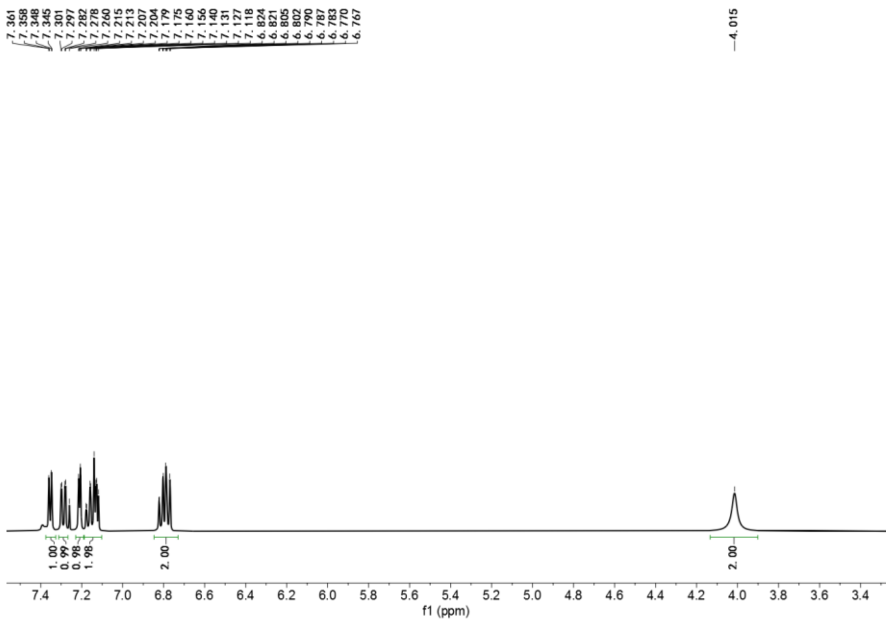

Figure S14. ^{1}H NMR spectrum of **BT**.

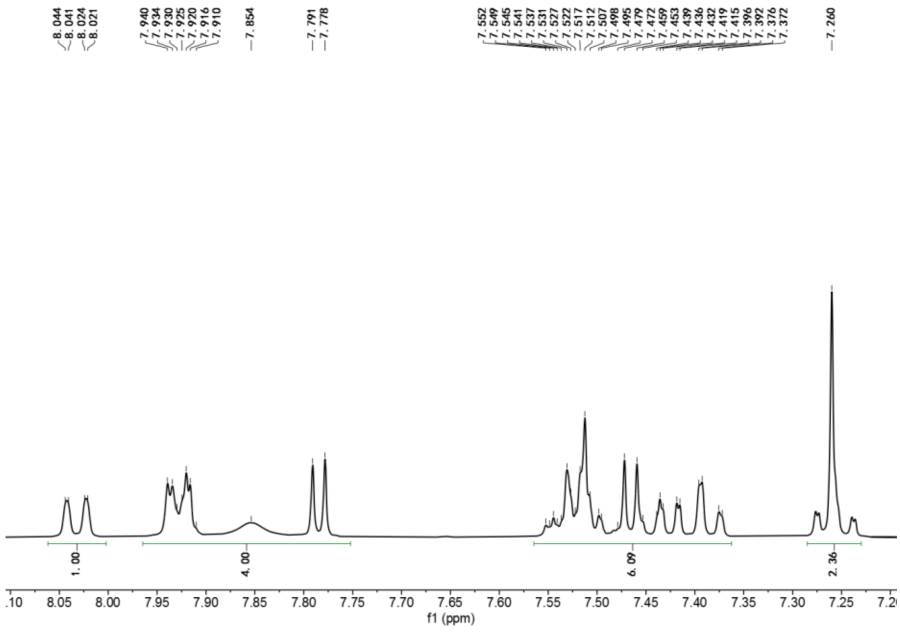

Figure S15. ^{1}H NMR spectrum of **BN-H**.

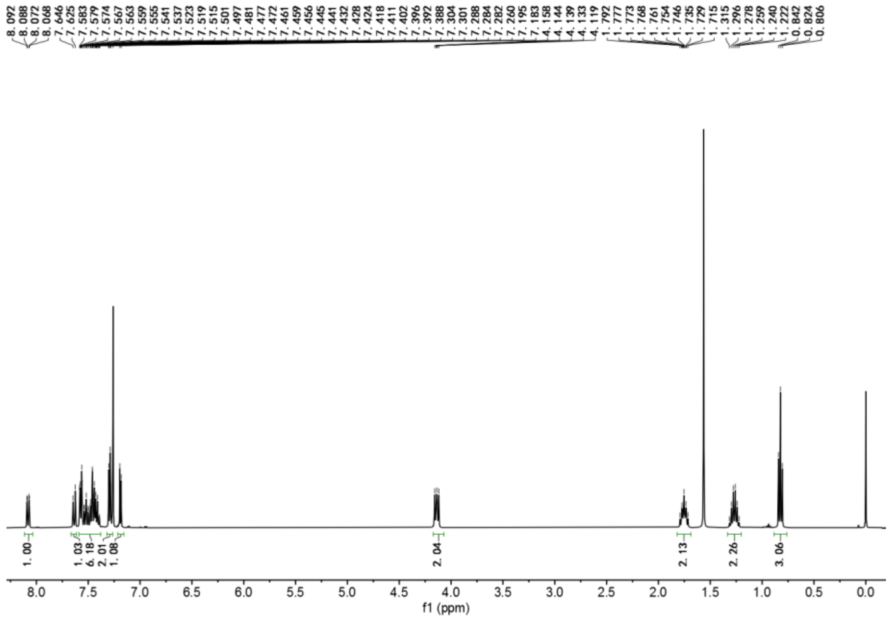

Figure S16. ^{1}H NMR spectrum of **BN-C4**.

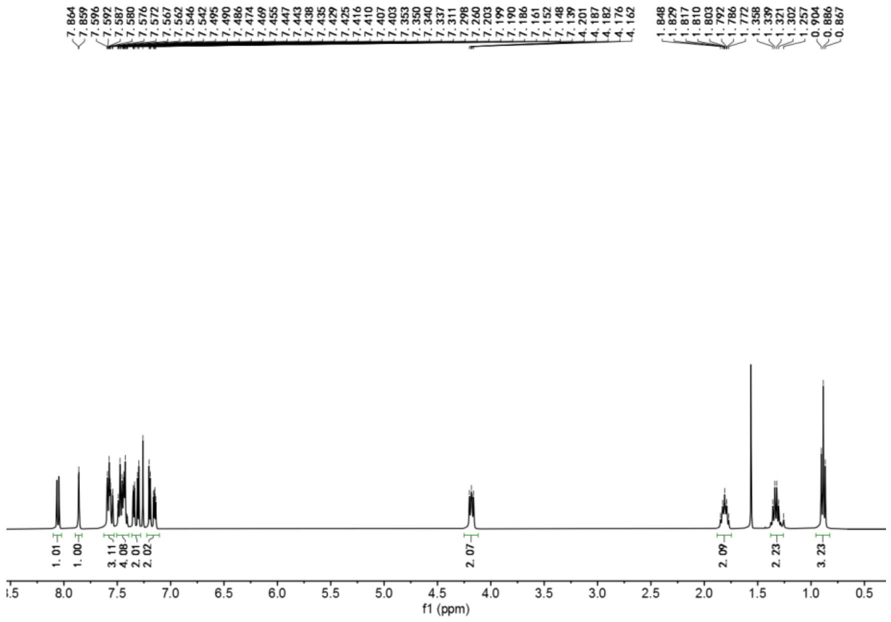

Figure S17. ^{1}H NMR spectrum of **BNT-C4**.

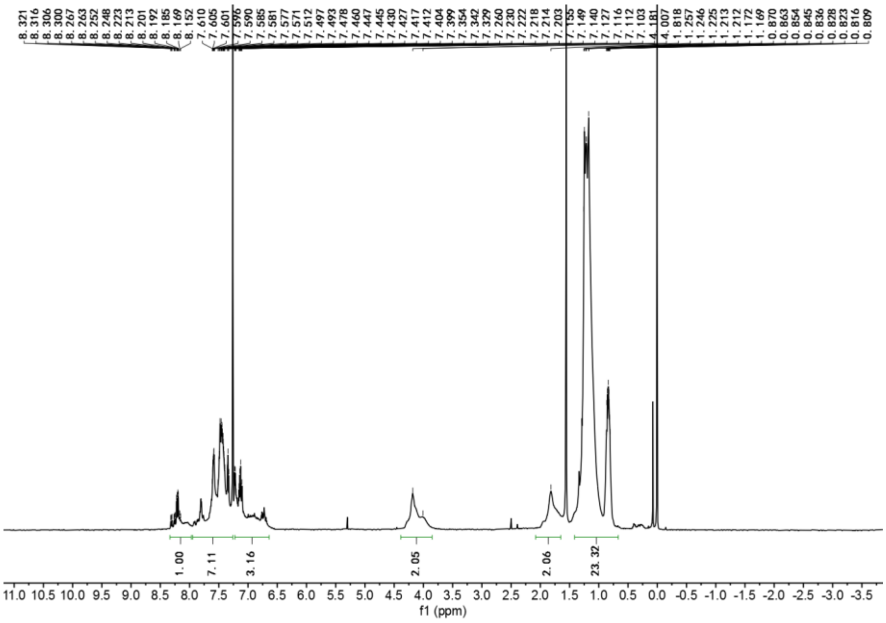

Figure S18. ^{1}H NMR spectrum of **P-BNT**.

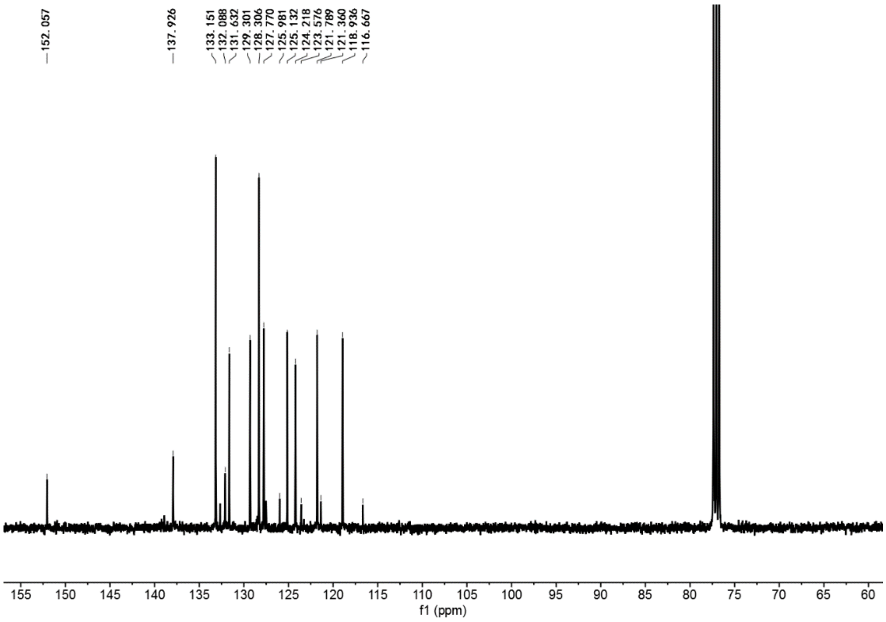

Figure S19. ^{13}C NMR spectrum of **BN-H**.

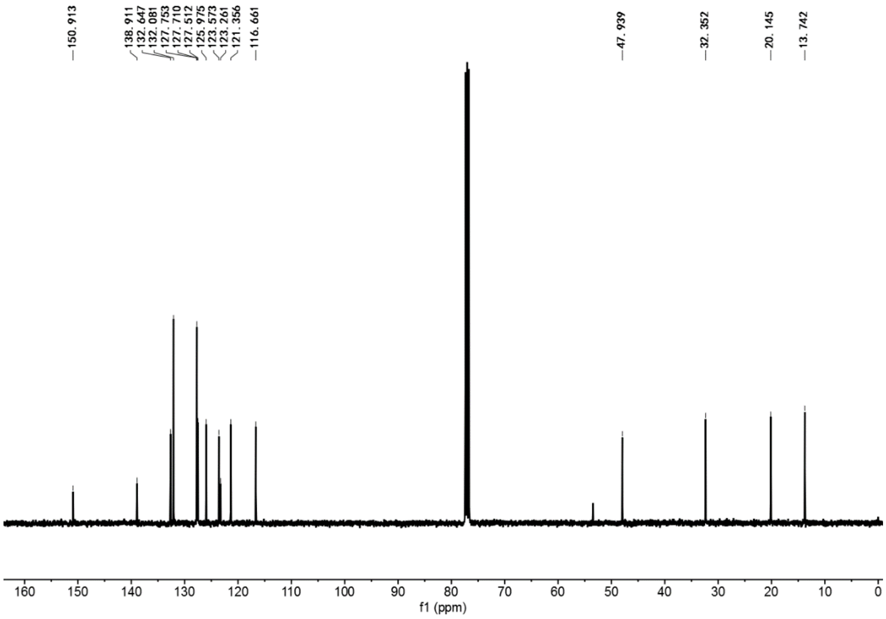

Figure S20. ^{13}C NMR spectrum of **BN-C4**.

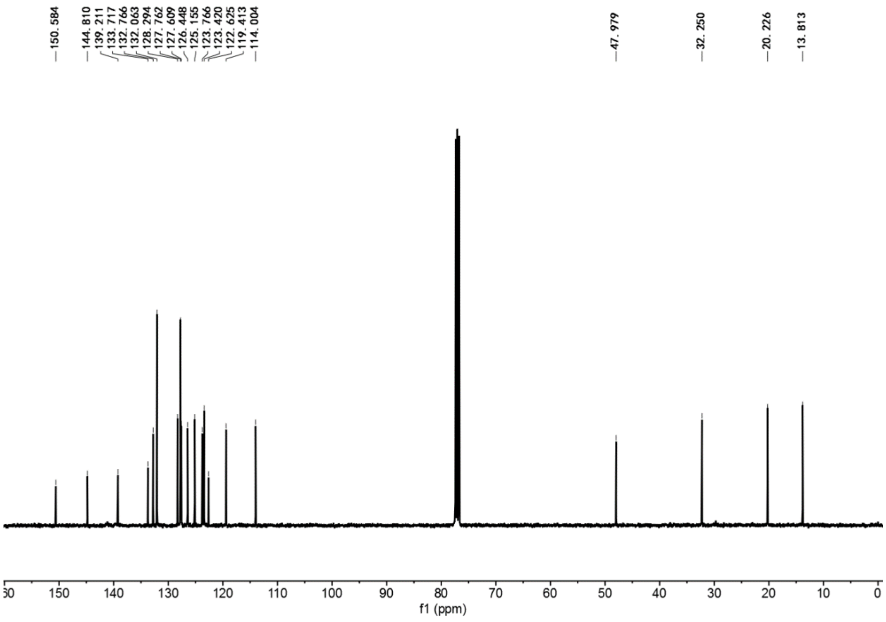

Figure S21. ^{13}C NMR spectrum of **BNT-C4**.

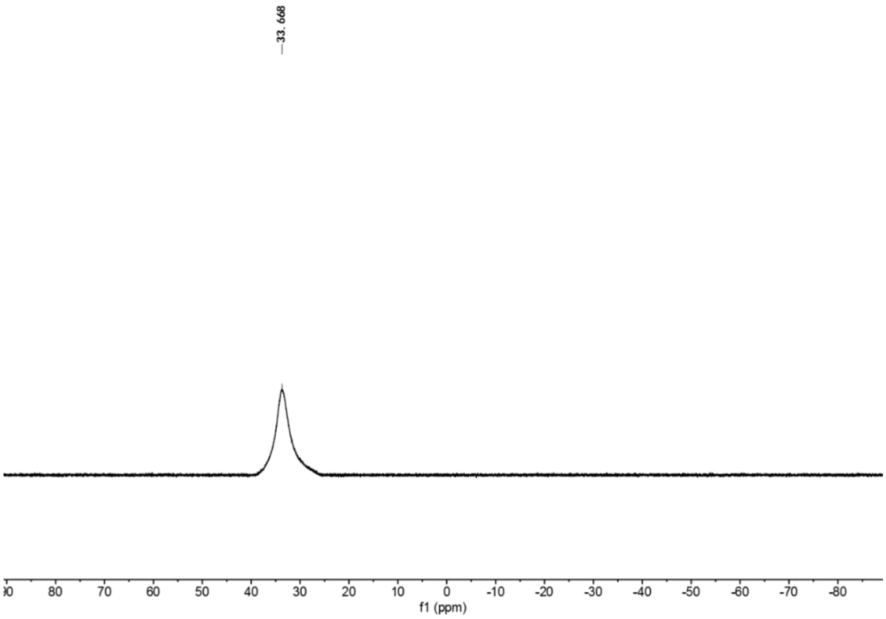

Figure S22. ^{11}B NMR spectrum of **BN-H**.

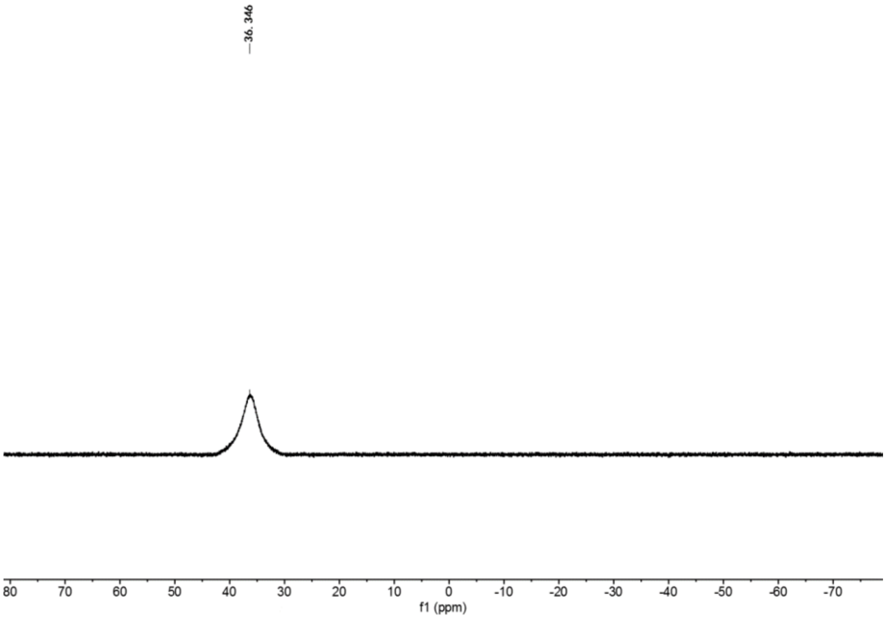

Figure S23. ^{11}B NMR spectrum of **BN-C4**.

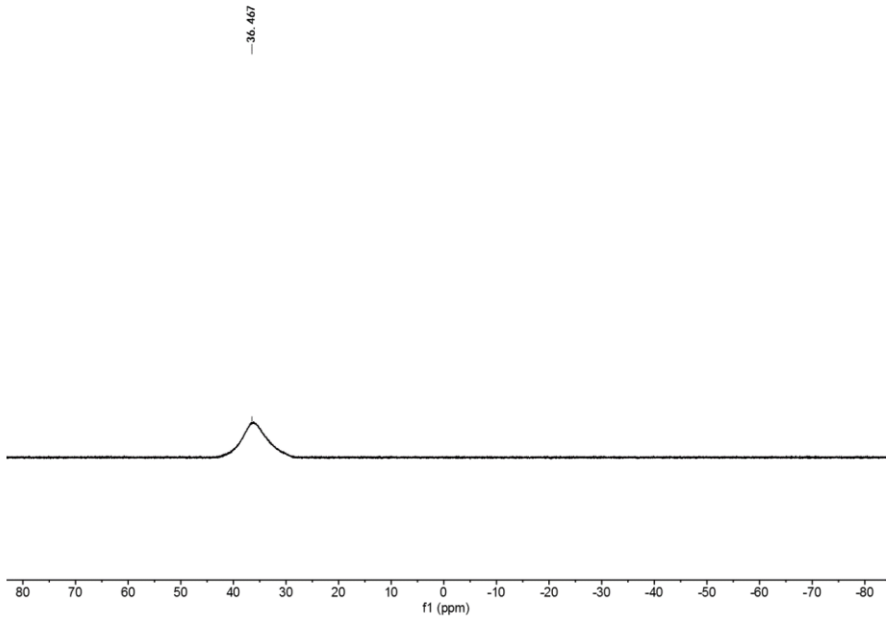

Figure S24. ^{11}B NMR spectrum of **BNT-C4**.

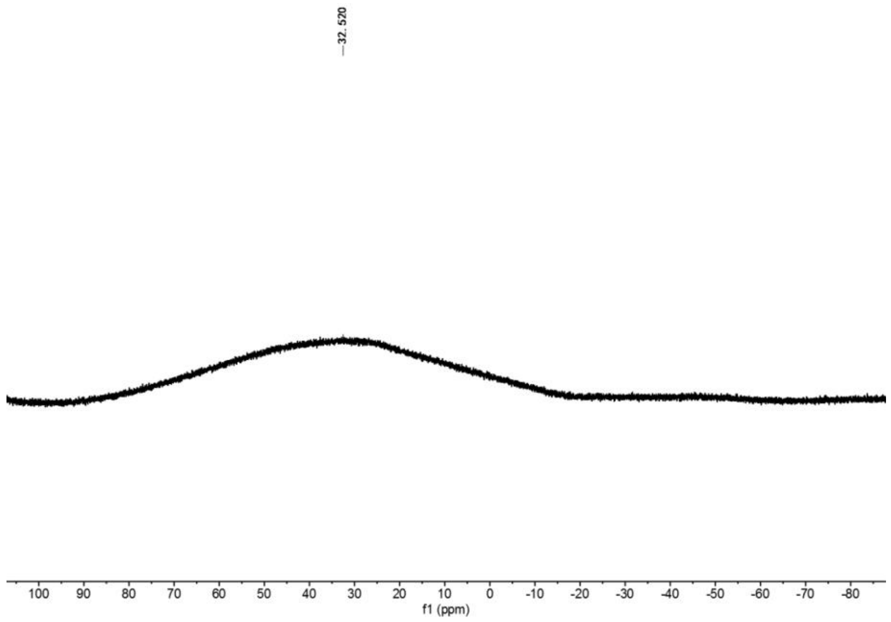

Figure S25. ^{11}B NMR spectrum of **P-BNT**.

6. References

[1] M. Frisch, G. Trucks, H. Schlegel, G. Scuseria, M. Robb, J. Cheeseman, G. Scalmani, V. Barone, B. Mennucci, G. Petersson, et al. Gaussian 09, Revision A.02; Gaussian, Inc.: Wallingford CT, 2009.

[2] L. Li, P. Gao, M. Baumgarten, K. Müllen, N. Lu, H. Fuchs, L. Chi, *Adv. Mater.* **2013**, 25, 3419–3425.

[3] F. Zhang, C. Di, N. Berdunov, Y. Hu, X. Gao, Q. Meng, H. Sirringhaus, D. Zhu, *Adv. Mater.* **2013**, 25, 1401–1407.

[4] M. Mirza, J. Wang, D. Li, S. A. Arabi, C. Jiang, *ACS Appl. Mater. Interfaces.* **2014**, 6, 5679–5684.

[5] H.W. Zan, W.W. Tsai; Y. Lo, Y.M. Wu, Y.S. Yang, I*EEE Sensors Journal.* **2012**, 594–601.

[6] A. Kalita, S. Hussain, A. H. Malik, Ni. V. V. Subbarao, P. K. Iyer, *J. Mater. Chem. C*, **2015**, **3**, 10767–10774.

[7] J. W. Jeong, Y. D. Lee, Y. M. Kim, Y. W. Park, J. H. Choi, T. H. Park, C. D. Soo, S. M. Won, I. K. Han, B. K. Ju, *Sensors and Actuators B: Chemical.* **2010**, 146,40–45.

[8] X. Xiao, X. F. Cheng, X. Hou, Ji. H. He, Q. F. Xu, H. Li, N. J. Li, D. Y. Chen, J. M. Lu, *Small.* **2017**, 13, 1602190.

[9] C. Kumar, G. Rawat, H. Kumar, Y. Kumar, R. Prakash, S. Jit, *Organic Electronics*. **2017**, 48, 53–60.
